# Low food and nutrition literacy (FNLIT): a barrier to dietary diversity and nutrient adequacy in school age children

**DOI:** 10.1186/s13104-020-05123-0

**Published:** 2020-06-12

**Authors:** Azam Doustmohammadian, Nasrin Omidvar, Nastaran Keshavarz-Mohammadi, Hassan Eini-Zinab, Maryam Amini, Morteza Abdollahi, Zeinab Amirhamidi, Homa Haidari

**Affiliations:** 1 Department of Nutrition Research, Department of Community Nutrition, National Nutrition and Food Technology Research Institute (WHO Collaborating Center) and Faculty of Nutrition Sciences and Food Technology, Shahid Beheshti University of Medical Sciences, Tehran, Iran; 2 Department of Community Nutrition, National Nutrition and Food Technology Research Institute (WHO Collaborating Center) and Faculty of Nutrition Sciences and Food Technology, Shahid Beheshti University of Medical Sciences, Tehran, Iran; 3 School of Public Health and Safety, Shahid Beheshti University of Medical Sciences, Tehran, Iran; 4 Department of Nutrition Research, National Nutrition and Food Technology Research Institute (WHO Collaborating Center) and Faculty of Nutrition Sciences and Food Technology, Shahid Beheshti University of Medical Sciences, Tehran, Iran

**Keywords:** Food and Nutrition Literacy, Dietary diversity, Nutrient adequacy, School age children, Iran

## Abstract

**Objective:**

This study aims to assess the relationship between Food and Nutrition Literacy (FNLIT) and dietary diversity score (DDS); FNLIT and nutrient adequacy (NAR%, MAR%) in school-age children in Iran.

**Results:**

This cross-sectional study was undertaken on 803 primary school students in Tehran, Iran. Socio-economic, as well as three 24-h dietary recalls were collected through interviewing students and their mothers/caregivers. FNLIT was measured by a self-administered locally designed and validated questionnaire. Low level of FFNL was significantly associated with higher odds of low DDS (OR = 2.19, 95% CI 1.32–3.62), the first tertile of fruit diversity score (OR = 3.88, 95% CI 2.14–6.99), and the first tertile of dairy diversity score (OR = 9.60, 95% CI 2.07–44.58). Low level of IFNL was significantly associated with probability of lower meat diversity score (OR = 1.73, 95% CI 1.07–2.81). Low level of FLL was also significantly associated with probability of lower DDS (OR = 1.81, 95% CI 1.11–2.94), dairy diversity score (OR = 2.01, 95% CI 1.02–3.98), and meat diversity score (OR = 2.14, 95% CI 1.32–3.45).Low FNLIT and its subscales were associated with higher odds of low level of NAR of protein, calcium, vitamin B3, B6, B9, as well as the probability of lower level of MAR.

## Introduction

Prevalence of Non-Communicable Diseases (NCDs) is a public health problem in Iran [1]. Therefore, an important priority for the health sector is capacity building within the public to prevent NCDs by empowering people to take control of the determinants of their health and disease [2]. Health literacy is considered as one of the most important skills to enable individuals to control health determinants [3]. Due to wide scope of health issues and because of the growing prevalence of diet-related chronic diseases [4], evidence suggest that one should consider health literacy more specifically [5]. As a result, food literacy/nutrition literacy has been proposed and conceptualized.

Studies have found that food literacy/nutrition literacy can have a critical role in shaping children’s dietary behaviors [6] and enabling them to have healthy food choices [7, 8]. In Iran, nutrition transition has taken place due to urbanization and rapid socio-economic changes and have resulted in a tendency toward a more westernized dietary pattern, especially among children and adolescents [9]. This general shift in children’s diet is characterized as low consumption of fruit and vegetables, fiber-rich foods and dairy products [10], as well as high consumption of fatty, sugary and convenience foods [11].

Considering today’s children food environment, improving food and nutrition literacy provides an opportunity for them to acquire appropriate skills and act more consciously [12]. Identifying the relation between food and nutrition literacy and children dietary intake is important for the development of effective prevention and management strategies in these age groups [6]. Yet, there is a gap of published evidence in this area. Therefore, This study aims to assess the relationship between Food and Nutrition Literacy (FNLIT) and dietary diversity score (DDS); as well as FNLIT and nutrient adequacy (NAR%, MAR%) in school-age children in Tehran, Iran.

## Main text

### Methods

#### Study design and setting

This school-based cross-sectional survey was performed using a multistage random cluster sampling design. The study was conducted in Tehran the capital city of Iran from November to January 2016. The STROBE (Strengthening The Reporting of Observational Studies in Epidemiology) study conduct [13] and participant flow is outlined in (Additional file 1: Fig. S1).

#### Study participants

The sample included 803 primary school students (419 boys and 384 girls) aged 10–12 years (power study 88%; response rate = 89.2%) from different socio-economic districts. Selected students and their parents were invited to take part in the study after signing a consent letter.

#### Data collection

##### Food and nutrition literacy assessments

Food and nutrition literacy (FNLIT) was measured by a valid self-administered questionnaire [14]. The questionnaire consisted of 4 true–false and 42 likert-type items within two cognitive and skill domains. Cognitive domain included two sub-scales: understanding food and nutrition information (UFNI, 10 items) and nutritional health knowledge (NHK, 5 items). Skill domain consisted of four sub-scales: functional food and nutrition literacy (FFNL, 10 items), interactive food and nutrition literacy (IFNL, 7 items), food choice literacy (FCL, 6 items) and critical food and nutrition literacy (CFNL, 4 items). Finally, Food label literacy (FLL) was evaluated by 4 true–false items. According to ROC analysis, three levels for FNLIT was low (≤ 51), medium (> 51– < 74) and high (≥ 74), where the FNLIT score ranged from 25.8 to 96.8 [15]. The questionnaire was completed by students.

##### Dietary intake assessments

Three 24-h dietary recalls (2 week-days and one holiday) was collected by interviewing the students and their mothers and/or other caregivers. To identify misreporting, BMR (basal metabolic rate) was estimated by using the equations published by Schofield (16). An individual’s daily food intake was considered under-reported, if EI (energy intake) to BMR ratio (EI/BMR) was less than 1.14, or over-reported if it was higher than 2.5 (17). Over- and under-reporters were excluded from the study as ‘misreporters’. After data cleaning and excluding all outliers and diet recall misreporters (170 students), 493 students remained for statistical analysis (see Additional file 1: Fig.S1). The characteristics of subjects excluded did not differ significantly from those remained in the study. Dietary intake adequacy-The nutrient adequacy ratio (NAR%)- was calculated for energy and 11 nutrients. NAR was calculated as the intake of a nutrient divided by the recommended intake for that nutrient (RNI) [16]. Mean adequacy ratio (MAR %) was calculated as a measure of the adequacy of overall diet, where MAR is the sum of each NAR (truncated at 100%) divided by the number of nutrients (excluding energy and protein) [17].

Dietary Diversity Score (DDS)–DDS was calculated as part of the pyramid serving database that was categorized into 23 broad food groups. Each of the 5 broad food categories received a maximum diversity score of 2 of the 10 possible score points. To be counted as a “consumer” for any of the food groups categories, a respondent needed to consume at least one-third serving at any time during a 3-day survey period [18].

#### Covariates

A number of evidence-based covariates [19–22] were considered in the study. Anthropometric measurements were taken [23]. BMI-Z-score for age and sex was calculated by WHO AnthroPlus, 2007 [24]. Physical activity was measured through interviewing children by the locally validated version of Child and Adolescent International physical activity questionnaires [25, 26]. Household food security status was measured using a locally validated 18-item USDA’s Household Food Security Survey Module through face-to-face interview with mothers [27, 28]. Demographic and socio-economic characteristics were collected by a questionnaire through interviewing with students and verified by their mothers and/or caregivers thereafter.

#### Statistical analysis

Data were presented as means and standard deviations for continuous variables and frequencies and percentages for categorical variables. Chi square test was used for analysis of general characteristics of participants. Independent sample *t* test was used to evaluate the differences between continuous variable between two sexes. Multinomial logistic regression analysis adjusting for confounding factors of the lower two tertiles compared with the higher tertile of MAR, NAR and DDS was conducted. All statistical analysis was performed using SPSS 21.0 (SPSS Inc., Chicago, Illinois, US).

### Results

#### Characteristics of the study participants

Table 1, summarizes background characteristics of the studied children by FNLIT status.

**Table 1:** General characteristics of 10–12 years old students by FNLIT (Food and Nutrition Literacy) status in Tehran

Demographic characteristics	Total	Low FNLIT ≤ 51	Moderate/high FNLIT > 51	p value*
	N (%)	N (%)	N (%)	
Sex	800			0.46
Male	419 (52.4)	52 (55.9)	367 (51.9)	
Female	381 (47.6)	41 (44.1)	340 (48.1)	
Grade	800			0.26
Fifth	413 (51.6)	43 (46.2)	370 (52.3)	
Sixth	387 (48.4)	50 (53.8)	337 (47.7)	
Birth order	798			0.15
< 2	437 (54.8)	44 (47.8)	393 (55.7)	
≥ 2	361 (45.2)	48 (52.2)	313 (44.3)	
Father age tertile (year):	790			0.47
T1:30–40	300 (38.0)	38 (42.7)	262 (37.4)	
T2:41–45	265 (33.5)	25 (28.1)	240 (34.2)	
T3: ≤ 46	225 (28.5)	26 (29.2)	199 (28.4)	
Mother age tertile (year):	794			0.20
T1:23–35	288 (36.3)	34 (37.0)	254 (36.2)	
T2:36–40	303 (38.2)	41 (44.6)	262 (37.3)	
T3: ≥ 41	203 (25.6)	17 (18.5)	186 (26.5)	
Ethnicity	797			0.99
Fars	441 (55.3)	50 (54.3)	391 (55.5)	
Azeri	228 (28.6)	27 (29.3)	201 (28.5)	
Fars-Azeri	56 (7.0)	7 (7.6)	49 (7.0)	
Other	72 (9.0)	8 (8.7)	64 (9.1)	
School type	800			0.78
Public	725 (90.6)	85 (91.4)	640 (90.5)	
Private	75 (9.4)	8 (8.6)	67 (9.5)	
Family size	797			0.140
≤ 3	160 (20.1)	15 (16.3)	145 (20.6)	
4	465 (58.3)	50 (54.3)	415 (58.9)	
≥ 5	172 (21.6)	27 (29.3)	145 (20.6)	
Fa ther education	789			0.45
Illiterate to ≤ 5 years	85 (10.8)	12 (13.5)	73 (10.4)	
6–9 years up to diploma	395 (50.1)	47 (52.8)	384 (49.7)	
Associate’s degree or higher	309 (39.2)	30 (33.7)	279 (39.9)	
Mother education	794			0.001*
Illiterate up to ≤ 5 years	86 (10.8)	13 (14.1)	73 (10.4)	
6–9 years up to diploma	461 (58.1)	66 (71.7)	395 (56.3)	
Associate’s degree or higher	247 (31.1)	13 (14.1)	234 (33.3)	
Father job position	690			0.11
Worker	106 (13.6)	18 (20.5)	88 (12.8)	
Employee/clerk	327 (41.4)	36 (40.9)	291 (42.2)	
High rank employee	139 (17.9)	10 (11.4)	129 (18.7)	
Retired	20 (2.6)	4 (4.5)	16 (2.3)	
Self-manager	186 (23.9)	20 (22.7)	166 (24.1)	
Mother employment	794			0.58
Working	630 (79.3)	75 (81.5)	55 (79.1)	
Housewife	164 (20.7)	17 (18.5)	147 (20.9)	
Other income source of family members	799			0.98
No	752 (94.1)	84 (90.3)	668 (94.6)	
Yes	47 (5.9)	9 (9.7)	38 (5.4)	
House ownership status	799			0.55
Owner	427 (53.4)	49 (52.7)	378 (53.5)	
Tenant	262 (32.8)	32 (34.4)	230 (32.6)	
Mortgage	35 (4.4)	6 (6.5)	29 (4.1)	
Other	75 (9.4)	6 (6.5)	69 (9.8)	
Financial support source	799			0.94
No	781 (97.7)	91 (97.8)	690 (97.7)	
Yes	18 (2.3)	2 (2.2)	16 (2.3)	
Physical activity tertile (MET.h/day)	787			0.31
Mean *T1*: 33	260 (33.0)	36 (39.1)	224 (32.2)	
Mean *T2*: 38.37	262 (33.3)	25 (27.2)	237 (34.1)	
Mean *T3*: 47.71	265 (33.7)	31 (33.7)	234 (33.7)	
Z score for BMI	800			0.16
Thinness	15 (1.9)	4 (4.3)	11 (1.6)	
Normal	381 (47.6)	47 (50.5)	334 (47.2)	
Overweight	213 (26.6)	19 (20.4)	194 (27.4)	
Obese	191 (23.9)	23 (24.7)	168 (23.8)	
HH food security status	746			0.33
FS	560 (75.1)	59 (68.6)	501 (75.9)	
FI without hunger	131 (17.6)	19 (22.1)	112 (17.0)	
FI with hunger	55 (7.4)	8 (9.3)	47(7.1)	

*HH* household, *FS* food secure, *FI* food insecure

*Significant at p < 0.05 for x^2^ tests

#### Food and nutrition literacy (FNLIT)

**Fig. 1:**
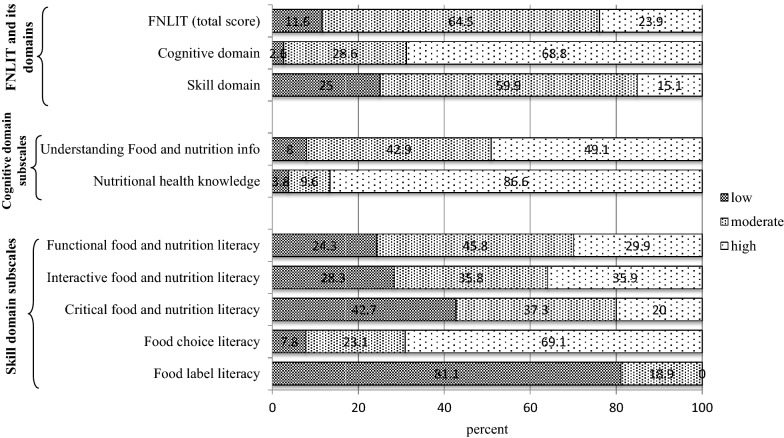
Food and nutrition literacy status in 10–12 years old students in Tehran

FNLIT status of the participants is presented in Fig. 1. Almost 25% of students had low scores in FNLIT skill domain, while the majority scored moderate to high in cognitive domain (97.4%). Among subscales of FNLIT skill domain, high proportion of students had low scores in CFNL (42.7%) as well as food label literacy (81.1%). However, they scored better in FCL and only 7.8% scored low.

#### Dietary intake adequacy

MAR and NAR of certain nutrients by sex are presented in (Additional file 2: Fig.S2). The multinomial-adjusted odds ratio (95% CI) of the lower two tertiles compared with the highest tertile of MAR and NAR of certain nutrients are presented in (Additional file 3: Table S1). Low levels of FNLIT was significantly associated with odds of lower level of NAR of protein (OR = 2.02, 95% CI 1.02–8.95). Low level of UFNI significantly increased the probability of having lower level of MAR and NAR of vitamin B9 (OR = 2.91, 95% CI 1.03–8.23, OR = 2.98, 95% CI 1.04–8.51 respectively). Low level of FFNL was significantly associated with odds of lower levels of MAR and NAR of vitamin B6 (OR = 3.12, 95% CI 1.38–7.05, OR = 2.30, 95% CI 1.10–4.83, respectively) and with odds of lower two tertiles compared with higher tertile of NAR of calcium (OR = 2.98, 95% CI 1.46–6.11, OR = 2.34, 95% CI 1.16–4.76 respectively). Low level of FCL was significantly associated with probability of lower level of NAR of vitamin B3 (OR = 3.65, 95% CI 1.05–12.69) and low level of FLL was significantly associated with probability of lower level of NAR of calcium (OR = 2.28, 95% CI 1.16–4.49).

#### Dietary diversity

Unadjusted and adjusted multinomial logistic regression of the lower two tertiles compared with the higher tertile of DDS are presented in Table 2. Adjusting for mother education, slightly improved the odds of certain DDS groups, including DDS, fruit diversity score and dairy diversity score for FFNL, and protein foods diversity score for IFNL.

**Table 2:** Adjusted Odd Ratios (95% CI) of Dietary Diversity Score (DDS) for FNLIT scale and its subscales in 10–12 years students in Tehran (n = 493)

Dietary diversity score (DDS)																
Groups	DDS				Fruits				Dairies				Protein foods			
Models	Unadjusted relative risk ratio (95% CI)		Adjusted relative risk ratio (95% CI)		Unadjusted relative risk ratio (95% CI)		Adjusted relative risk ratio(95% CI)		Unadjusted relative risk ratio (95% CI)		Adjusted relative risk ratio (95% CI)		Unadjusted relative risk ratio (95% CI)		Adjusted relative risk ratio (95% CI)	
	T1	T2	T1	T2	T1	T2	T1	T2	T1	T2	T1	T2	T1	T2	T1	T2
Low FFNL																
Yes	2.19* (1.32, 3.62)	0.85 (0.48, 1.52)	2.25* (1.35, 3.76)	0.88 (0.49, 1.56)	3.88* (2.14, 6.99)	1.68^*^(1.06, 2.67)	3.90* (2.17, 7.00)	1.72^*^(1.08, 2.74)	9.60^*^(2.07, 44.58)	6.88^*^(1.45, 32.65)	9.80^*^(2.08, 46.11)	7.13^*^(1.48, 34.26)	0.98 (0.55, 1.74)	0.98 (0.58, 1.64)	0.98 (0.55, 1.74)	0.98 (0.58, 1.64)
No	*1*	1	*1*	1			*1*	*1*	*1*	*1*	*1*	*1*	1	1	1	1
Low IFNL																
Yes	0.96 (0.61, 1.52)	0.94 (0.58, 1.52)	0.98 (0.62, 1.54)	0.94 (0.58, 1.52)	0.83 (0.47, 1.49)	0.97 (0.65, 1.46)	0.83 (0.46, 1.49)	0.98 (0.65, 1.48)	1.05 (0.47, 2.33)	1.02 (0.44, 2.33)	1.06 (0.47, 2.36)	1.02 (0.44, 2.35)	0.91 (0.52, 1.58)	1.73^*^(1.07, 2.81)	0.92 (0.53, 1.59)	1.75^*^(1.08, 2.84)
No	1	1	1	1	1	1	1	1	1	*1*	1	1	1	*1*	1	*1*
Low FLL																
Yes	1.81^*^(1.11, 2.94)	1.32 (0.81, 2.15)	1.71^*^(1.05, 2.79)	1.33 (0.81, 2.16)	1.07 (0.58, 1.99)	1.39 (0.90, 2.15)	1.06 (0.57, 2.05)	1.32 (0.85, 2.37)	2.01^*^(1.02, 3.98)	2.16^*^(1.03, 4.50)	1.98 (0.81, 4.86)	2.05^*^(1.01, 4.36)	1.64 (0.97, 2.76)	2.14^*^(1.32, 3.45)	1.61 (0.95, 2.73)	2.06^*^(1.27, 3.34)
No	1	1	*1*	1					*1*	*1*	1	*1*			1	*1*

Multinomial logistic regression, adjusted for mother education

Only those variables that were significantly associated with FNLIT and its subscales were reported

*FNLIT* Food and Nutrition Literacy, Cognitive subscales including: *UFNI* Understanding Food and Nutrition Literacy, *NHK* Nutritional Health Knowledge, Skill subscales including, *FFNL*, Functional Food and Nutrition Literacy, *IFNL* Interactive Food and Nutrition Literacy, *FCL* Food Choice Literacy, *CFNL* Critical Food and Nutrition Literacy, *FLL* Food Label Literacy

*Significant at p < 0.05

## Discussion

The present study showed that low food and nutrition literacy may be a barrier to dietary diversity and nutrient adequacy in school age children. Previous research has shown that high food literacy is associated with increased consumption of fruits and vegetables [29, 30]. Children and adolescents who assisted in preparing meals were more likely to engage in food preparation-related behaviors such as buying fresh vegetables, writing grocery lists and preparing meals with chicken, fish or vegetables [31]. Besides, FNLIT skills encompass the ability to obtain factual dietary information and develop an understanding of factors that can enhance or inhibit nutritional health [5] and may promote diet diversity and nutrient adequacy through improving understanding of available food and nutrition information and adherence to the dietary guidelines [5]. Low level of FNLIT was significantly associated with probability of lower level of meat group diversity (as major sources of protein and iron) and NAR of calcium, the two major limiting nutrients in the Iranian’s diet [32].

FLL was one of the weakest areas among the studied children. However, the question is to what degree food labeling is an appropriate approach and what type of information and labeling approaches can be helpful for children. Previous reports have identified food label reading as one of the key areas to improve food choices and dietary intakes in children [33]. In Iran, mandatory nutrition labeling for food products has recently been initiated while there are still gaps in its regulatory policy [34]. Besides, this concept and its application has not been entered in public education programs, including schools curricula and textbooks to empower individuals in making healthy food choices [35]. This may explain low scores observed in the study population with regard to questions on food labels.

The considerable proportions of students with low food and nutrition literacy in skill domain imply a gap in food and nutrition skill development in primary school curriculums in the country. This is not a surprise as based on content analysis of primary school textbooks in Iran, nutritional content of school textbooks were mainly theoretical and impractical [35]. This leads to many students with high food and nutrition knowledge, but limited skills in their dietary practices. Even though, nutrition knowledge has been identified as essential for behavior change [36], knowledge alone is generally not sufficient to produce sustained behavior change in complex behaviors [36]. Therefore, FNLIT subscale-based interventions should be designed to improve students’ skills and nutrition behaviors. For example, the results provide a reminder of the need to support improvements in food choices and dietary behaviors by helping young people to develop skills such as those required to interpret food labeling.

## Conclusion

This study demonstrates that low level of FNLIT is associated with nutritional inadequacy in school-age childeren and low DDS and may play an important role in shaping their dietary intake. FNLIT is expected to have effect on one’s ability to assess information when choosing foods, comprehend food labels, and apply dietary recommendations. Therefore, it is important for educators and program planners to assess and enhance FNLIT of young people. Stakeholders, including policy makers, food manufacturers, health providers, educators, and businesses should also play their roles so as to achieve a bigger impact on future generation.

## Limitation

The current study has several limitations.

- First, there was a possibility of recall bias and social desirability bias.
- Second, due to its cross-sectional design, our study was not able to establish any cause-effect relationship of food and nutrition literacy on children’s dietary intakes.
- Third, the excluded data was quite large, due to lack of access to parents by phone, although we found no significant difference in characteristics of dropout subjects with those remained in the study.

## Supplementary information

**Additional file 1: Fig. S1.** STROBE study conduct and participant flow of the stud**y.**

**Additional file 2: Fig. S2.** The nutrient adequacy ratio percent of certain nutrients by sex in 10–12 years students in Tehran (n = 493).

**Additional file 3: Table S1.** Adjusted Odd Ratios (95% CI) of nutrient adequacy ratio (MAR%/NAR%) of certain nutrients for FNLIT scale and its subscales in 10–12 years students in Tehran (n = 493).

## Data Availability

The dataset supporting the conclusions of this article can be made available upon request after approval by the authors.

## Abbreviations

FNLIT: Food and nutrition literacy
NCDs: Non-communicable diseases
ROC: Receiver operating characteristic
HEI: Healthy eating index
BMR: Basal metabolic rate
NAR: Nutrient adequacy ratio
RNI: Recommended intake for that nutrient
MAR: Mean adequacy ratio
DDS: Dietary diversity score
BMI: Body mass index
METs-h/day: Metabolic equivalents hour/day
UFNI: Understanding Food and Nutrition Information
NHK: Nutritional health knowledge
FFNL: Functional food and nutrition literacy
IFNL: Interactive food and nutrition literacy
FCL: Food choice literacy
CFNL: Critical food and nutrition literacy
FLL: Food label literacy
