# Supplementary material for: Low food and nutrition literacy (FNLIT): a barrier to dietary diversity and nutrient adequacy in school age children
Source: BMC Res Notes. 2020 Jun 12;13:286. doi: 10.1186/s13104-020-05123-0 (PMC7291429; doi:10.1186/s13104-020-05123-0)
Supplement: Supplementary file 1 — Additional file 1: Fig. S1. STROBE study conduct and participant flow of the study. [file 13104_2020_5123_MOESM1_ESM.docx]

900 invited persons

Participants without written consents (n=97)

803 participants with complete consents

Response rate=89.2%

Participants without food and nutrition literacy records (n=3)

Participants without complete demographic questionnaire (n=54)

Participants without 24 hour dietary recall (n=86)

- 803 participants with completed demographic data
- 800 participants with completed FNLIT data
- 663 participants with completed 24-hour dietary recalls

Exclusion of outliers and misreports (n=170)

Children involved in statistical analysis (n=493)

**Fig. S1** STROBE study conduct and participant flow of the study
