# Supplementary material for: Low food and nutrition literacy (FNLIT): a barrier to dietary diversity and nutrient adequacy in school age children
Source: BMC Res Notes. 2020 Jun 12;13:286. doi: 10.1186/s13104-020-05123-0 (PMC7291429; doi:10.1186/s13104-020-05123-0)
Supplement: Supplementary file 2 — Additional file 2: Fig. S2. The nutrient adequacy ratio percent of certain nutrients by sex in 10–12 years students in Tehran (n = 493). [file 13104_2020_5123_MOESM2_ESM.docx]

**Fig. S2** The nutrient adequacy ratio percent of certain nutrients by sex in 10-12 years students in Tehran (n=493)

*Significant at p<0.001 for t-test

** Significant at p<0.05 for t-test
