# Supplementary material for: Low food and nutrition literacy (FNLIT): a barrier to dietary diversity and nutrient adequacy in school age children
Source: BMC Res Notes. 2020 Jun 12;13:286. doi: 10.1186/s13104-020-05123-0 (PMC7291429; doi:10.1186/s13104-020-05123-0)
Supplement: Supplementary file 3 — Additional file 3: Table S1. Adjusted Odd Ratios (95% CI) of nutrient adequacy ratio (MAR%/NAR%) of certain nutrients for FNLIT scale and its subscales in 10–12 years students in Tehran (n = 493). [file 13104_2020_5123_MOESM3_ESM.docx]

Table S1: Adjusted^b^ Odd Ratios (95% CI) of nutrient adequacy ratio (MAR%/NAR%) of certain nutrients for FNLIT scale and its subscales in 10-12 years students in Tehran (n=493) ^c^

| **Nutrient adequacy ratio (MAR%/NAR%) of certain nutrients** | | | | | | | | | | | | |
| --- | --- | --- | --- | --- | --- | --- | --- | --- | --- | --- | --- | --- |
| **NAR** | **MAR** | | **NAR pro** | | **NAR B_9_** | | **NAR B_6_** | | **NAR B_3_** | | **NAR calcium** | |
|  | T1 | T2 | T1 | T2 | T1 | T2 | T1 | T2 | T1 | T2 | T1 | T2 |
| **Low FNLIT ^a^** |  |  |  |  |  |  |  |  |  |  |  |  |
| **Yes** | 1.33  (0.38,4.54) | 0.80  (0.29,2.15) | 2.47  (0.61,10.02) | **2.02**  **(1.02,8.95)** | 0.85  (0.31,2.35) | 0.88  (0.33,2.32) | 1.49  (0.50,4.44) | 1.37  (0.52,3.58) | 0.79  (0.23,2.66) | 0.48  (0.17,1.37) | 1.24  (0.43,3.60) | 0.81  (0.26,2.39) |
| **No** | 1 | 1 | 1 | **1** | 1 | 1 | 1 | 1 | 1 | 1 | 1 | 1 |
| **Low UFNI** |  |  |  |  |  |  |  |  |  |  |  |  |
| **Yes** | 2.94  (0.82,10.44) | **2.91**  **(1.03,8.23)** | 0.32  (0.08,1.29) | 0.38  (0.14,1.06) | **2.98**  **(1.04,8.51)** | 1.44  (0.52,3.99) | 0.84  (0.28,2.54) | 1.03  (0.39,2.69) | 1.26  (0.40,3.93) | 0.91  (0.32,2.57) | 0.34  (0.12,1.01) | 0.45  (0.17,1.20) |
| **No** | 1 | **1** | 1 | 1 | **1** | 1 | 1 | 1 | 1 | 1 | 1 | 1 |
| **Low FFNL** |  |  |  |  |  |  |  |  |  |  |  |  |
| **Yes** | **3.12**  **(1.38,7.05)** | 1.72  (0.91,3.24) | 2.33  (0.98,5.52) | 1.67  (0.84,3.30) | 1.75  (0.89,3.42) | 1.03  (0.58,2.03) | **2.30**  **(1.10,4.83)** | 1.32  (0.70,2.47) | 0.61  (0.27,1.36) | 0.78  (0.39,1.57) | **2.98**  **(1.46,6.11)** | **2.34**  **(1.16,4.76)** |
| **No** | 1 | 1 | 1 | 1 | 1 | 1 | **1** | 1 | 1 | 1 | **1** | **1** |
| **Low FCL** |  |  |  |  |  |  |  |  |  |  |  |  |
| **Yes** | 0.83  (0.23,2.96) | 0.75  (0.27,2.09) | 0.84  (0.18,3.83) | 1.13  (0.37,3.41) | 0.81  (0.26,2.47) | 1.16  (0.43,3.08) | 2.10  (0.67,6.54) | 0.97  (0.35,2.69) | **3.65**  **(1.05,12.69)** | 2.35  (0.78,7.08) | 0.73  (0.43,1.25) | 0.89  (0.53,1.49) |
| **No** | 1 | 1 | 1 | 1 | 1 | 1 | 1 | 1 | 1 | **1** | 1 | 1 |
| **Low FLL** |  |  |  |  |  |  |  |  |  |  |  |  |
| **Yes** | 0.83 (0.37,4.38) | 0.98 (0.54,1.77) | 0.94 (0.41,2.16) | 1.15 (0.61,2.19) | 0.92 (0.47,1.82) | 0.84 (0.47,1.50) | 1.31 (0.63,2.71) | 1.41 (0.77,2.57) | 0.59 (0.28,1.27) | 0.89 (0.44,1.79) | 1.11  (0.58,2.11) | **2.28**  **(1.16,4.49)** |
| **No** | 1 | 1 | 1 | 1 | 1 | 1 | 1 | 1 | 1 | 1 | 1 | **1** |

^a^ FNLIT, Food and Nutrition Literacy; Cognitive subscales including: UFNI, Understanding Food and Nutrition Literacy; NHK, Nutritional Health Knowledge; Skill subscales including; FFNL, Functional Food and Nutrition Literacy IFNL, Interactive Food and Nutrition Literacy; FCL, Food Choice Literacy; CFNL, Critical Food and Nutrition Literacy; FLL, Food Label Literacy.

^b^ Multinomial logistic regression, adjusted for sex, school status (governmental and nongovernmental), grade, birth rank, family size, ethnicity, parents’ age, parents’ education, father’s job position, mother’s employment, Other income source of family members, house ownership status, financial support source, physical activity, household food security, weight status and calorie intake

^c^ Only those variables that were significantly associated with FNLIT and its subscales were reported.

*Significant at p<0.05
